# Effects of roads on giant panda distribution: a mountain range scale evaluation

**DOI:** 10.1038/s41598-018-37447-0

**Published:** 2019-02-01

**Authors:** Ke He, Qiang Dai, Xianghui Gu, Zejun Zhang, Jiang Zhou, Dunwu Qi, Xiaodong Gu, Xuyu Yang, Wen Zhang, Biao Yang, Zhisong Yang

**Affiliations:** 1 Chengdu Institute of Biology, Chinese Academy of Sciences, Chengdu, 610041 China; 2 School of Life Sciences, Guizhou Normal University, Guiyang, 550001 China; 3 Key Laboratory of Southwest China Wildlife Resources Conservation (Ministry Of Education), China West Normal University, Nanchong, 637002 China; 4 Chengdu Research Base of Giant Panda Breeding, Sichuan Key Laboratory of Conservation Biology for Endangered Wildlife, Chengdu, 610086 China; 5 Sichuan Station of Wild life survey and Management, Chengdu, 610082 China; 6 Sichuan Provincial Institute of Forestry Survey and Planning, Chengdu, 610082 China

## Abstract

Few studies have focused on the mountain ranges scale effects of roads on wildlife. This lack of data could lead to an underestimation of the negative impact of roads on animal populations. We analyzed a dataset that included 74.4% of the giant panda population and covered 78.7% of the global giant panda habitat to estimate road-effect zones for major roads, and to investigate how these major roads influenced the distribution of giant pandas on a mountain range spatial scale. We found that the density of giant panda signs was significantly decreased by proximity to major roads. The effect zone reached 5,000 m from national roads and 1,500 m from provincial roads. Structural equation model analysis revealed that the strongest negative impact of major roads on giant pandas was via the reduction of nearby forest cover. The results should provide a better understanding of the impact of anthropogenic infrastructure and regional economic development on wildlife, thus providing a basis for conservation policy decisions. We suggest that the environmental impact assessment of proposed roadways or further researches on road ecological effects should expand to a larger scale and consider the possible habitat degradation caused by road access.

## Introduction

Roads are one of the largest artificial man-made structures on the planet. Roadways have allowed human activity, and the accompanying negative impacts on the ecosystem, to reach nearly every region of the earth’s surface^1^. Roads exert various negative effects on wildlife^2,3^, including road mortality^4,5^, road avoidance^6^, the barrier effect^7^ and habitat degradation^8^. The road-effect zone is defined as the distance from the road, over which significant ecological effects can be detected^1,9^.

On the landscape scale, habitat degradation caused by human activities, including the indirect effects on wildlife, can extend outward over a much wider distance^10^ than that from the direct effects of roads^1,11^. Caribou density, for example, was decreased within a 5 km road-effect zone near a highway^12^. In the Amazon, nearly 95% of all deforestation occurs within 5.5 km of roads^10^. Nevertheless, too few quantitative studies exist concerning the effects of roads on wildlife at larger spatial scales (e.g., greater than 10 km from roads), this could lead to an underestimation of the negative impact of roads on wildlife.

During the 18th and 19th centuries, giant pandas were distributed over a wide region of east Asia^13^. The subsequent distribution retreat is believed to have been caused by both global climate change^14,15^ and anthropogenic disturbances^14–16^. As roads are a major source of these disturbances, understanding the sizes of road-effect zones is essential for road planning and decision making in conservation policies. Gong, *et al*.^17^ evaluated the effect zone for hiking trails in the Qinling Mountains, they found that the giant panda was significantly less likely to be found within 500 and 1,000 m from hiking trails. Although several research studies have found that giant pandas avoid roads^18–21^, the size of the road-effect zone is still unknown. The lack of available, data has made statistical analysis difficult, especially for small heterogeneous regions.

This study aimed to assess the extent of road-effect zones for major roads (national roads and provincial roads), and to investigate how those major roads influence the distribution of the giant panda on a mountain range scale. The data set included 74.4% of the giant panda population (1387 individuals) and covered 78.7% of the global giant panda habitat (20272 km^2^)^22^. We hypothesized that the distribution of giant pandas near major roads would be affected more by the indirect effects of roads (e.g. via changing the land cover or increasing the amount of human disturbance) than by the direct effects of roads. The results of this study will be important for both national and local road planning in the context of giant panda conservation. This study is also noteworthy in that understanding how roads influence the giant panda distribution may lead to more effective mitigation efforts.

## Results

**Figure 1:**
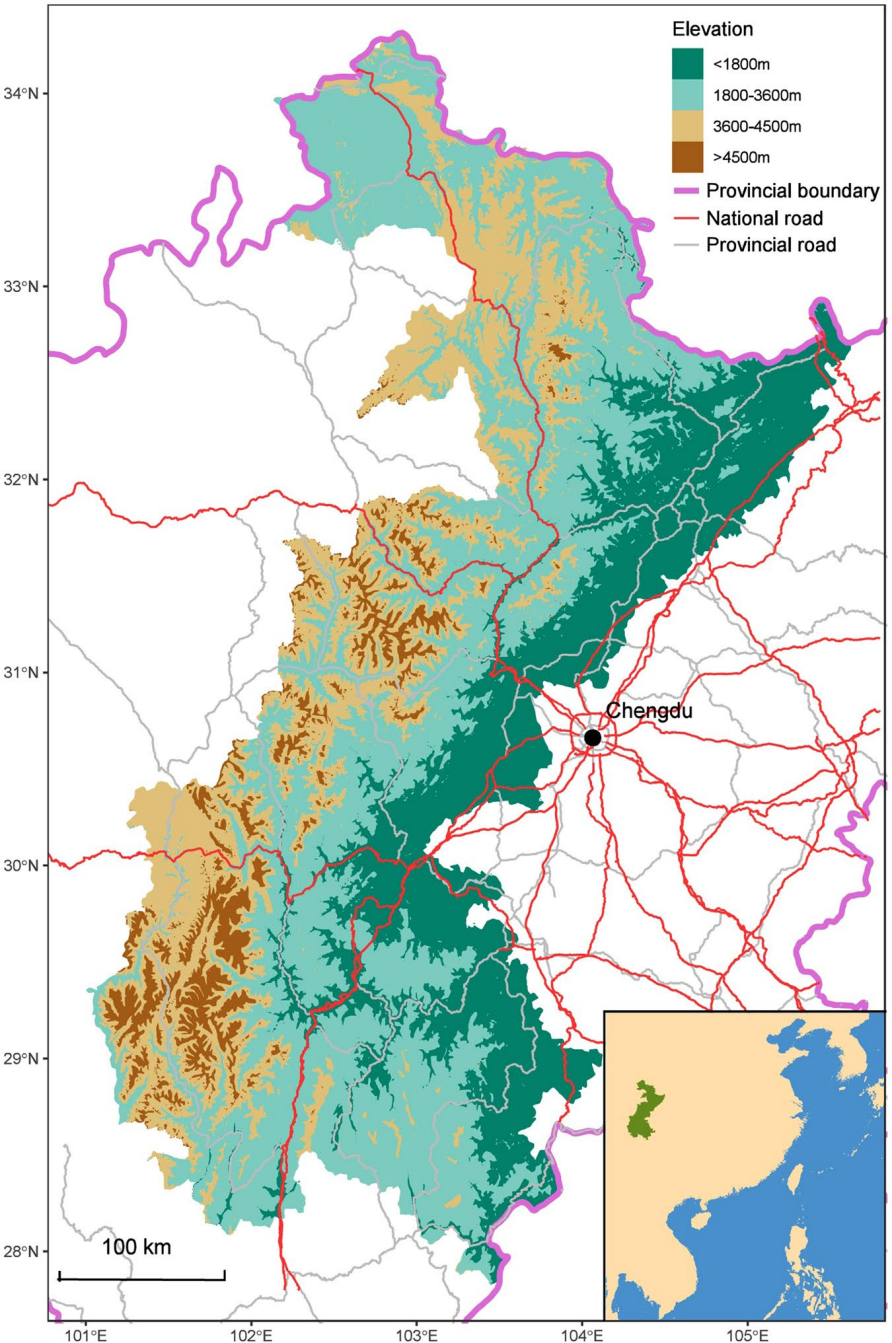
Study area. Map was created with package “ggplot2” in R environment^49,50^.

**Figure 2:**
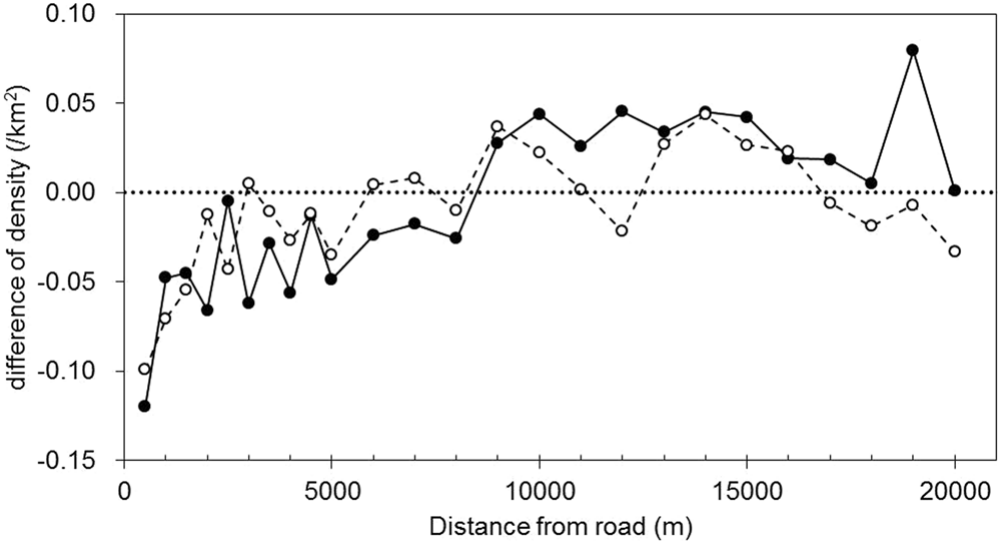
The difference in densities of giant pandas signs (densities of signs in buffers near roads minus those in random copies) within 20000 m from roads. The solid line with black circles represents national roads, and the dashed line with open circles represents provincial roads. The dotted line indicates no difference between the densities of signs near roads and random buffers^52^.

Overall, 4258 records of giant panda presence were observed in five mountain ranges covering 117113 km^2^, during the Fourth National Giant Panda Survey (NGPS4) carried out from 2011 to 2013. To determine whether giant pandas avoid the areas surrounding roads, we compared the densities of giant panda signs near roads against those in random portions of the study areas (Fig. 1). The densities of giant panda signs at distances from national roads between 0 and 5000 m were significantly lower than those from random portions, at 6 of the 10500 m intervals (Fig. 2, Table 1). There were no significant differences between the densities of giant panda signs beyond 5000 m. For provincial roads, significant differences in densities of signs were only found within 1500 m.

**Table 1:** Results from a paired Mann-Whitney U test comparing the densities of giant panda signs in buffers around roads and random copies for both national and provincial roads.

Distance from road (m)	National roads				Provincial roads			
	Difference of sign density	*U*	n	*p*	Difference of sign density	*U*	n	*p*
500	−0.120	0	29	0.011^*^	−0.099	0	52	<0.001^**^
1000	−0.048	4	29	0.054	−0.070	20	50	0.012^*^
1500	−0.045	9	34	0.018^*^	−0.054	42	70	0.006^*^
2000	−0.066	14	31	0.015^*^	−0.012	83	65	0.211
2500	−0.005	5	32	0.572	−0.043	44	70	0.012
3000	−0.062	13	36	0.041^*^	0.005	73	66	0.612
3500	−0.028	2	38	0.089	−0.010	62	70	0.254
4000	−0.056	5	39	0.040^*^	−0.026	54	65	0.149
4500	−0.013	14	41	0.312	−0.012	85	66	0.351
5000	−0.048	0	31	0.018^*^	−0.035	15	53	0.111
6000	−0.024	33	59	0.066	0.004	319	123	0.230
7000	−0.017	27	58	0.104	0.008	286	118	0.543
8000	−0.026	60	59	0.349	−0.010	357	119	0.325
9000	0.028	124	59	0.955	0.037	310	106	0.890
10000	0.044	180	62	0.988	0.022	236	112	0.939
11000	0.026	86	54	0.831	0.002	263	98	0.381
12000	0.045	112	54	0.956	−0.022	340	95	0.175
13000	0.034	101	54	0.882	0.027	284	84	0.858
14000	0.045	102	49	0.891	0.044	368	92	0.942
15000	0.042	89	48	0.953	0.027	321	87	0.859
16000	0.019	82	55	0.899	0.023	412	90	0.729
17000	0.019	125	59	0.778	−0.006	274	84	0.457
18000	0.005	83	49	0.630	−0.019	213	80	0.348
19000	0.080	94	48	0.975	−0.007	218	84	0.197
20000	0.001	60	46	0.224	−0.033	162	76	0.117

^*^p < 0.05, and **p < 0.05.

We used structural equation models (SEMs) to quantify the direct (e.g., disturbance caused by traffic noise or light) and indirect (e.g., changes in vegetation cover or human population growth caused by roads) effects of roads on the density of giant panda signs. The final accepted SEM showed a good fit with to the data for both national roads (RMSEA < 0.05; *χ*^2^ test, *χ*^2^ = 5.297, *df* = 3, P > 0.1) and provincial roads (RMSEA < 0.05; *χ*^2^ test, *χ*^2^ = 5.349, *df* = 3, P > 0.1).

**Figure 3:**
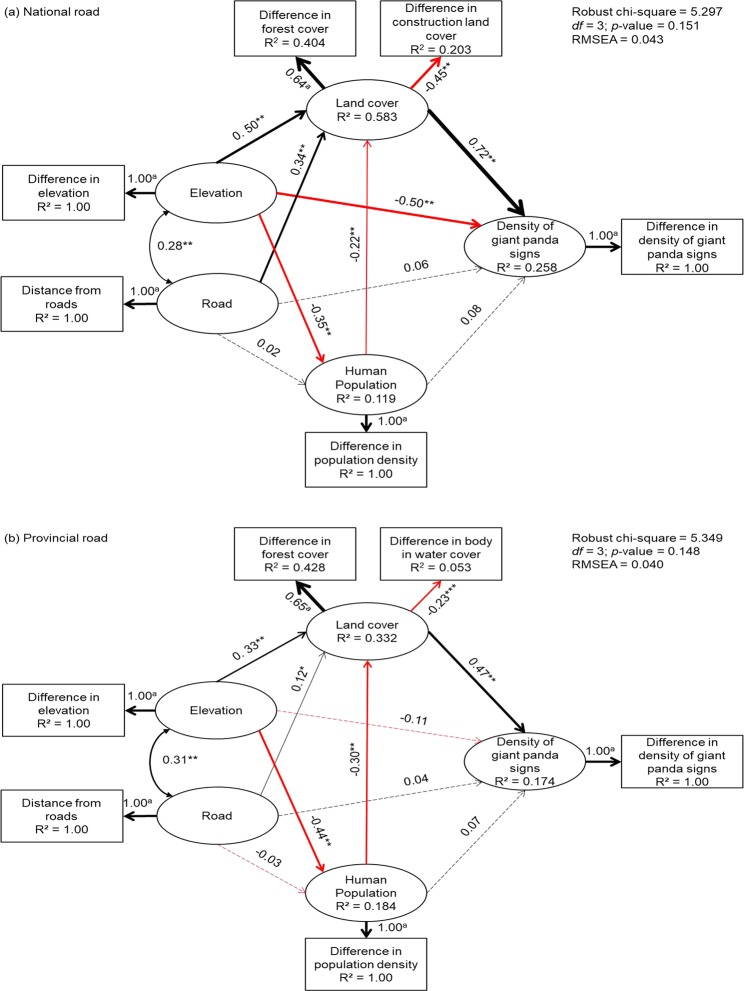
Structural equation models (SEMs) for the difference in densities of giant panda signs in buffers around roads and those in random buffers. (**a**) Difference of densities of giant panda signs near national roads: the land cover was defined by the proportion of forest and construction land. (**b**) Difference of densities of signs near provincial roads: the land cover was defined by forest cover and water cover. The values associated with the paths are the standardized path coefficients, and the thickness of black (positive) and red (negative) paths is proportional to the standardized path coefficients. Solid arrows indicate significant relationships (P-value < 0.05), and dashed arrows refer to non-significant paths. Double-headed arrows indicate covariance estimates. *Indicates results significant at the 0.05 level or lower, **indicates results significant at the 0.01 level or lower, and the superscript “a” indicates coefficients modeled as fixed parameters with no measurement error.

Near national roads, land cover was characterized by differences from random buffers in forest cover (0.64) and construction land cover (−0.45), and the structure of the SEM explained 25.8% of the total variance the density of in giant panda signs (R^2^ = 0.258) (Fig. 3a). The standardized path coefficients showed that land cover had the strongest direct effect (0.72) on the density of giant panda signs, followed by the elevation (−0.50). National roads showed no significant direct effects on the density of giant panda signs. Instead, national roads, as well as elevation and population, influenced the density of giant panda signs via their effects on land cover.

Our SEM explained 17.4% of the total variance in the density of giant panda signs near provincial roads (R^2^ = 0.174) (Fig. 3a). Land cover, the latent variable, was characterized by differences in forest cover (0.65) and body of water cover (−0.23). The results indicated that the density of giant panda signs was only directly influenced by land cover (0.47), while the land cover was controlled by elevation (0.33), roads (0.12) and the human population (−0.30).

## Discussion

We analyzed the effects of major roads on the distribution of giant pandas in five mountain ranges. Our study showed that the density of giant panda signs was significantly decreased by proximity to major roads. For national roads, the road-effect zone reached 5000 m away from the roads; for provincial roads, the zones reached 1500 m away from the roads. We found that the direct effect of roads on giant pandas may be weak at the mountain range scale, but roads may reduce the density of giant panda signs by reducing forest cover.

The road-effect zone for national roads (5000 m) was much wider than that for provincial roads (1500 m), and the road-effect zone for provincial roads was wider than that for hiking trails (1000 m)^17^. From the perspective of landscapes and socio-economic systems, major roads connect urban areas, towns, and villages by themselves or via the connecting minor branch roads. Anthropogenic disturbances, therefore, spread along major roads and expand to proximate regions. The effect of a major road extends far beyond the direct influences of the road itself^1^(i.e., such as noise^11,23^ and light pollution^3^). Major roads are generally constructed to connect residential areas; after completion, areas along the route are susceptible to increased economic development^24^. Generally, national roads connect larger urban areas, and have high traffic densities, thereby having a wider region of influence than provincial roads.

The giant panda is a forest species^25,26^ and is highly adapted to a specialized bamboo diet^27–29^, This explains suggests why our results indicate that the negative impacts of major roads on giant panda are mostly due to the roads’ effects on the forest cover. The effects of roads on forest cover extended much farther than the edge effects of roads^30^. Roads facilitate human access to natural resources, thus facilitating forest degradation^31,32^. A study carried out in Yunnan, southwest China, showed that forest habitats were lost at a drastic rate from 1991 to 2006, in regions adjacent to roads and urban areas, especially at relatively low altitudes (2025 m to 2301 m)^33^. Habitats at even lower altitudes may have been lost during earlier periods. Road access has facilitated logging in the mountain regions in the past, and even after a national logging ban was issued in China in 1998, roads continued to promote local economic growth. Roads have hastened the development of markets for tourism, which has exerted persistent disturbance on the forest habitat^34,35^. Substantial research has shown that the habitat of the giant panda have been lost or degraded due to increasing human activities, such as bamboo shoot collecting, livestock, firewood collecting and other forest-associated activities^36–38^. This indicates that reducing forest degradation facilitated by road access would be an effective way to mitigate the negative impacts of major roads on giant panda.

The results of the SEM showed that the direct effects of roads on the density of giant panda signs were fairly weak. However, this does not necessarily mean that direct effects have little impact on the giant panda. Direct effects, such as noise, light prolusion, and dust, seldom extend more than 1,000 m away from a road^1,3,23^, while giant panda signs within a wider buffer zone around major roads are already rare because of the change in land cover. Thus, it is possible that, the direct effects of roads are masked by the strength effect of the indirect effects.

Our results will be helpful to understand the impact of the anthropogenic infrastructure on wildlife populations. The analysis is relevant to regional economic development plans and conservation policy decisions. We suggest that the environmental impact assessment of proposed roadways and the corresponding mitigation methods should consider the possible habitat degradation caused by road access as well as the direct impacts of the road, in the form of noise, dust, and chemical pollution. We also suggest that further research on the ecological effects of roads should consider a larger scale. Given the fact that the sampling area of most studies is within 1 km of a road, this scale may be too small to show the full effects of a road (see but^10,12^).

## Method

### Study area

Our study area was located in Sichuan Province, western China, and covered 117113 km^2^. This region contains five mountain ranges (the Minshan Mountains, Qionglaishan Mountains, Daxiangling Mountains, Xiaoxiangling Mountains and Liangshan Mountains) that compose the most important habitat for the giant panda. The study area is part of the Hengduan Mountains biodiversity hotspot, one of Conservation International’s 35 Biodiversity hotspots in the world^39^, and therefore has significant value for conservation. More than 1000 km of paved roads, belonging to various classes, traverse the mountains and valleys in this area. National roads, which connect Chinese cities of economic and social significance, generally are wider and busier than provincial roads.

### Data and data sources

Giant panda records and road locations in the study area of Sichuan Province were provided by the NGPS4^22^, which was carried out from 2011 to 2013. In total 13681 1.4 × 1.4 km regular grid plots and 56 2.45 × 2.45 km regular grid plots were surveyed within the possible distribution region of the giant panda in Sichuan Province, and signs of the giant panda, including feces, fur, footprints, and paw marks, were recorded along with the location coordinate. Elevation data, taken from a digital elevation model with a 30-m resolution, were downloaded from the International Scientific & Technical Data Mirror Site, Computer Network Information Center, Chinese Academy of Sciences (http://www.gscloud.cn). Land cover data, including construction land, bodies of water and forest cover, were obtained from the Second National Forest Inventory and supplemented by the NGPS4 dataset. For the forest cover, we only included the natural forest, and the construction land only included buildings. The human population data were derived from the Sixth National Population Census^40^.

### Statistical analysis

Two different approaches were applied to determine the effects of roads on the distribution of the giant panda at the mountain range scale:

The density of giant panda signs is a relative index representing the intensity of utilization in a region by giant pandas. We compared the densities of giant panda signs near roads against those in randomly selected plots. The ring buffers from roads were segmented into small sections by 15 × 15 km grids, and, the random sections were created by shifting and rotating these grid sections to a new location within our study area. Ring buffers were created with radii spanning from 0 to 20000 m, calculated every 500 m up to 5000 m and every 1000 m above that. The densities of giant panda signs were calculated in both actual sections and random virtual sections. Only one record was kept for the computation if the distance between any pair of signs was less than 100 m.

Studies in various mountains showed that the giant panda prefers habitats at elevations roughly between 1500 m and 3500 m^26,41,42^. The data from the NGPS4 of Sichuan Province showed that 99% of giant panda signs were located between 1600 m and 3800 m. To exclude unsuitable habitat, regions higher than 3800 m or lower than 1600 m were clipped from the buffers and were included in the analysis.

The presence of roads can alter vegetation composition and structure^43^, but we did not directly use vegetation data in this analysis. As we determined the distances of effects imposed by roads in a large region of western China, the effects of vegetation were averaged across the heterogeneous landscape. Paired Mann-Whitney U tests were performed to compare the densities of giant panda signs in buffers near roads with those in random buffers.

**Figure 4:**
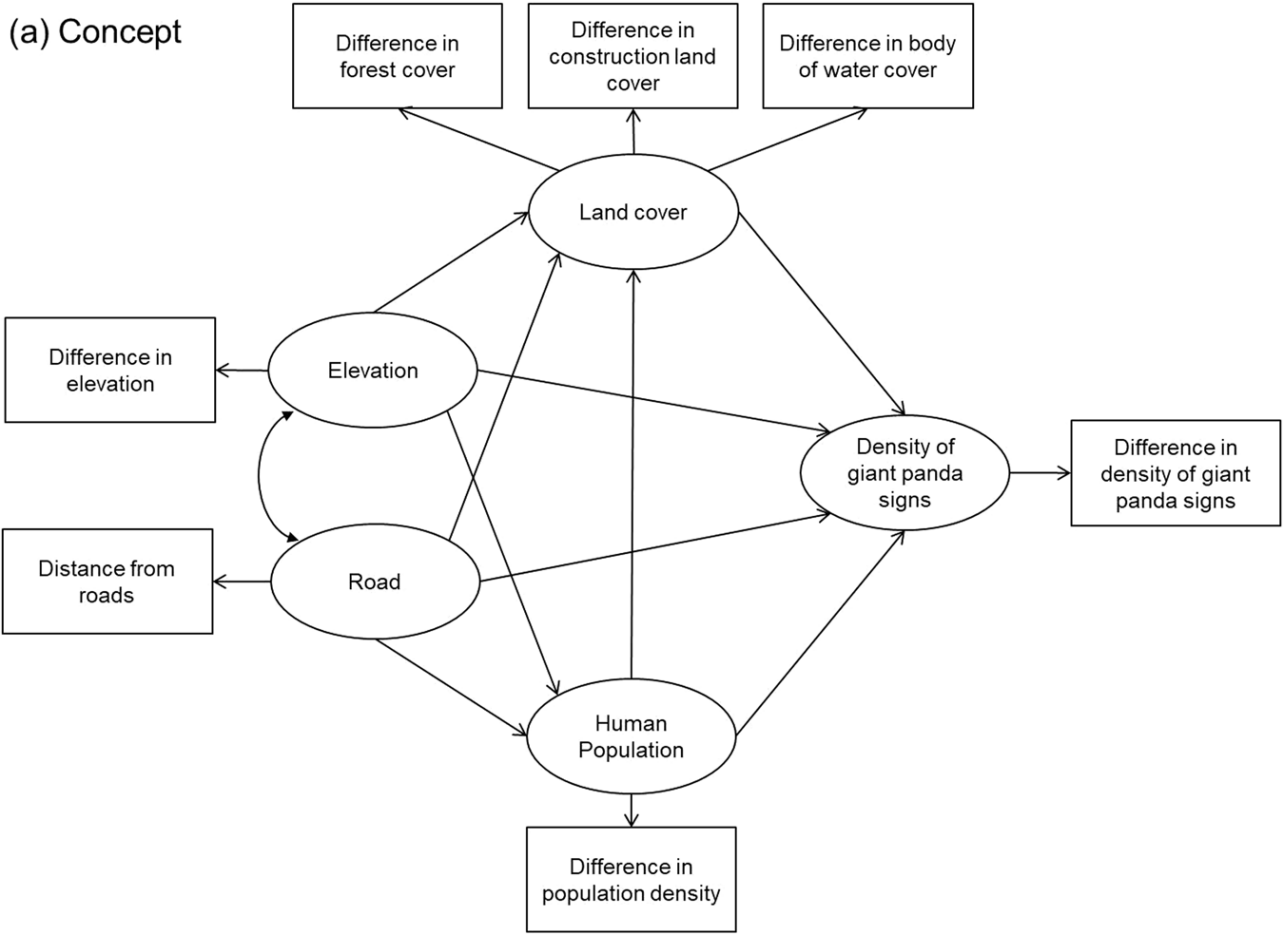
Conceptual model describing the expected associations between environmental factors and difference in densities of giant panda signs between buffers around roads and random buffers. The environmental factors include differences in land cover, defined as a latent variable with three indicator variables (proportion of forest, construction land and bodies of water), distance from roads, difference in average elevation, and differences in human population densities. Arrows represent possible path directions, and double-headed arrows indicate covariance estimates.

Structural equation models (SEMs) are multivariate statistical analyses of networks of causal relationships, SEMs are powerful at extracting direct and indirect effects^44–46^. SEMs allow rigorous estimation of indirect effects and tests of the overall fit of a complex, causal network of influence^47^. Based on expected pathways (Fig. 4), we developed conceptual SEMs so that the differences between densities of giant panda signs in buffers near roads and in random buffers would be predicted by distance from roads and differences in average elevation, human population density, and percentage of land cover. Latent factors representing land cover difference (proportion of forest, construction land and bodies of water) were assumed to be influenced by the distances from roads, differences in elevation, and human population densities (Fig. 4).

We fitted the SEMs using robust maximum likelihood, as some of the variables were not normally distributed^48^. The goodness of fit for each model was evaluated with the chi-square statistic and the root mean square error of approximation (RMSEA), where p-values higher than 0.05 for chi-square and lower than 0.05 for RMSEA indicated a good fit. To meet distributional requirements of linear relationships, the distances from roads were square root transformed. The initial concept models did not fit the data well for chi-square and RMSEA tests. Therefore the measured variables that indicated land use were stepwise removed to match the criteria of the chi-square and RMSEA for goodness of fit. The variable of forest cover was always kept, since forest was the most critical variable for the giant panda.

We performed all of the GIS analyses in R^49^, using the packages “rgdal”^50^ and “rgeos”^51^. We fit the SEMs using the R-package “lavaan”^48^.
